# Large-scale genomic analyses with machine learning uncover predictive patterns associated with fungal phytopathogenic lifestyles and traits

**DOI:** 10.1038/s41598-023-44005-w

**Published:** 2023-10-11

**Authors:** E. N. Dort, E. Layne, N. Feau, A. Butyaev, B. Henrissat, F. M. Martin, S. Haridas, A. Salamov, I. V. Grigoriev, M. Blanchette, R. C. Hamelin

**Affiliations:** 1 Department of Forest and Conservation Sciences, Faculty of Forestry, University of British Columbia, Vancouver, BC Canada; 2 School of Computer Science, McGill University, Montreal, QC Canada; 3 Pacific Forestry Centre, Canadian Forest Service, Natural Resources Canada, Victoria, BC Canada; 4 Department of Biotechnology and Biomedicine (DTU Bioengineering), Technical University of Denmark, 2800 Kgs. Lyngby, Denmark; 5 Department of Biological Sciences, King Abdulaziz University, Jeddah, Saudi Arabia; 6 Institut National de Recherche pour l’Agriculture, l’Alimentation et l’Environnement, Unité Mixte de Recherche Interactions Arbres/Microorganismes, Centre INRAE, Grand Est-Nancy, Université de Lorraine, Champenoux, France; 7 Lawrence Berkeley National Laboratory, U.S. Department of Energy Joint Genome Institute, Berkeley, CA USA; 8 Department of Plant and Microbial Biology, University of California Berkeley, Berkeley, CA USA; 9 Institut de Biologie Intégrative et des Systèmes (IBIS), Université Laval, Québec, QC Canada; 10 Département des Sciences du bois et de la Forêt, Faculté de Foresterie et Géographie, Université Laval, Québec, QC Canada

**Keywords:** Machine learning, Fungal genomics, Fungal pathogenesis

## Abstract

Invasive plant pathogenic fungi have a global impact, with devastating economic and environmental effects on crops and forests. Biosurveillance, a critical component of threat mitigation, requires risk prediction based on fungal lifestyles and traits. Recent studies have revealed distinct genomic patterns associated with specific groups of plant pathogenic fungi. We sought to establish whether these phytopathogenic genomic patterns hold across diverse taxonomic and ecological groups from the Ascomycota and Basidiomycota, and furthermore, if those patterns can be used in a predictive capacity for biosurveillance. Using a supervised machine learning approach that integrates phylogenetic and genomic data, we analyzed 387 fungal genomes to test a proof-of-concept for the use of genomic signatures in predicting fungal phytopathogenic lifestyles and traits during biosurveillance activities. Our machine learning feature sets were derived from genome annotation data of carbohydrate-active enzymes (CAZymes), peptidases, secondary metabolite clusters (SMCs), transporters, and transcription factors. We found that machine learning could successfully predict fungal lifestyles and traits across taxonomic groups, with the best predictive performance coming from feature sets comprising CAZyme, peptidase, and SMC data. While phylogeny was an important component in most predictions, the inclusion of genomic data improved prediction performance for every lifestyle and trait tested. Plant pathogenicity was one of the best-predicted traits, showing the promise of predictive genomics for biosurveillance applications. Furthermore, our machine learning approach revealed expansions in the number of genes from specific CAZyme and peptidase families in the genomes of plant pathogens compared to non-phytopathogenic genomes (saprotrophs, endo- and ectomycorrhizal fungi). Such genomic feature profiles give insight into the evolution of fungal phytopathogenicity and could be useful to predict the risks of unknown fungi in future biosurveillance activities.

## Introduction

The health of many natural and managed plant ecosystems is threatened by fungal plant pathogens, which often cause emerging infectious diseases that are difficult to mitigate once established^1–3^. Due to their perennial nature, trees are particularly vulnerable to non-native pathogens known as forest invasive alien species (FIAS), which can spread rapidly due to the lack of co-evolved resistance in native hosts, causing disease outbreaks across entire landscapes^4–7^. Biosurveillance–the systematic and cyclical process of collecting and analyzing surveillance data to detect and characterize disease outbreaks and inform subsequent management decisions–has become a crucial means for countries to reduce the threat of FIAS^7–10^. While current biosurveillance strategies enable regulatory agencies to identify known pathogens, they do not identify the specific ecological traits associated with disease outbreaks and also fail to monitor pathogens that have not yet been taxonomically identified or listed as potential threats. This missing information constitutes a blind spot in policies for pathogen regulation as most FIAS are not identified taxonomically until after they have successfully invaded ecosystems^6,11^. In light of these limitations, a genomics approach to biosurveillance that is focused on discovering the genomic ‘signatures’ that FIAS use to successfully invade novel ecosystems outside of their taxonomic identity has been proposed^8–10,12^. Indeed, a more genetics- and genomics-centered approach to plant pathogen management is an increasingly prevalent theme in recent research^13–16^.

An important question to be answered for genomics-based biosurveillance is whether there are genomic signatures associated with the lifestyles and traits of fungal plant pathogens. The success of FIAS, and of phytopathogenic fungi in general, hinges on their diverse trophic strategies, or lifestyles, which enable them to infect and colonize a variety of plant tissues. In addition to their lifestyles, fungal phytopathogens display diverse ecological traits such as host range and tissue specificity. These lifestyle and trait categories encompass generalizations about the pathogens that make up each group, including how they infect their hosts and spread disease through ecosystems. However, it is often challenging to assign species to discrete lifestyle categories due to the complex behaviours of many fungi and their ability to exhibit multiple lifestyles; this becomes particularly problematic when trying to understand the mechanisms of fungal pathogenicity and plant disease resistance^17,18^. Given the subjectivity in assigning pathogens to lifestyle and trait categories, there has been increased research to explore these categories at the genome level, especially given the increasing availability of whole genome sequences^19–21^. Additionally, the continued growth of online fungal genomic resources and databases such as FungiDB^22^, Ensembl Genomes^23^, NCBI RefSeq^24^, and MycoCosm^25^ are providing researchers with powerful resources to compare fungi with different lifestyles and traits at the genome scale.

Many recently published comparative genomics studies focus on groups of important plant-interacting fungi such as wood-decay fungi^26^, dark septate endophytes^27^, mycorrhizal fungi^28,29^, and phytopathogenic groups including *Colletotrichum*^30,31^, *Zymoseptoria*^32^, *Fusarium*^33^, and Dothideomycetes^21,34^ species. These large-scale analyses reveal genomic patterns that can help plant pathologists better understand fungal genome evolution and identify genes that may be central to phytopathogenicity. From a biosurveillance perspective, the discovery of distinct genomic signatures associated with phytopathogenic lifestyles or traits could help predict risk or impact of undescribed fungal pathogens by analyzing their gene content even before they have been taxonomically described^12^. Integrating genomic analyses into biosurveillance pipelines would allow regulatory agencies to predict the threat a pathogen poses to an ecosystem and respond accordingly.

Previous studies have demonstrated that there are lifestyle-related genomic signatures present in specific groups of fungal plant pathogens^19–21,30–35^. We sought to build on these findings in a larger and more diverse group of fungi spanning a subset of classes from the Ascomycota and Basidiomycota phyla to test a proof-of-concept for using predictive genomics in the biosurveillance of fungal plant pathogens. In addition to fungal lifestyles, we expanded our analyses to include important ecological traits associated with plant pathogens and relevant to the biosurveillance of FIAS. Our results revealed that both lifestyles and traits can be predicted from fungal genomes, and we were able to uncover genomic features influencing phytopathogenicity in fungi. Our findings have important implications for integrating predictive genomics into future fungal FIAS biosurveillance pipelines and, in the long term, the development of more effective management strategies.

## Results

### Phytopathogenic lifestyles and traits can be predicted from phylogenetic and genomic data

**Figure 1:**
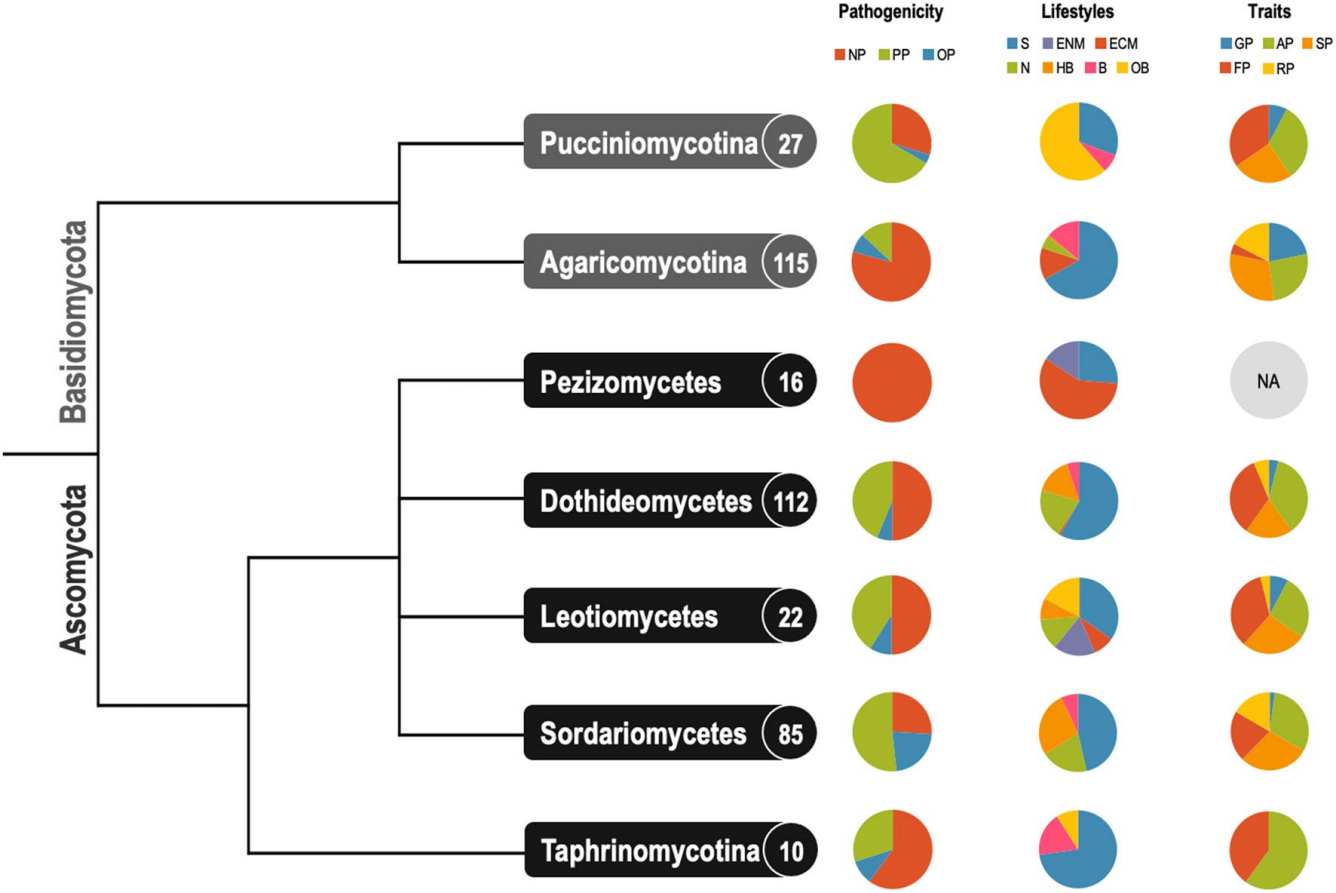
Phylogenetic groups (includes subphyla and classes) from FunLifeDB included in our genomic analyses with summaries of the categories represented for each group analysed. The tree shows the phylogenetic relationships between the groups as represented by MycoCosm (https://mycocosm.jgi.doe.gov/mycocosm/home). The circled number after each group name indicates the total number of genomes analysed for that group, and the subsequent pie charts indicate how many of those genomes belong to each pathogenic, lifestyle and trait category. *NP* non-pathogen, *PP* plant pathogen, *OP* other pathogen, *S* saprotroph, *ENM* endomycorrhizal, *ECM* ectomycorrhizal, *N* necrotroph, *HB* hemibiotroph, *B* biotroph, *OB* obligate biotroph, *GP* gymnosperm pathogen, *AP* angiosperm pathogen, *SP* stem pathogen, *FP* foliar pathogen, *RP* root pathogen.

**Figure 2:**
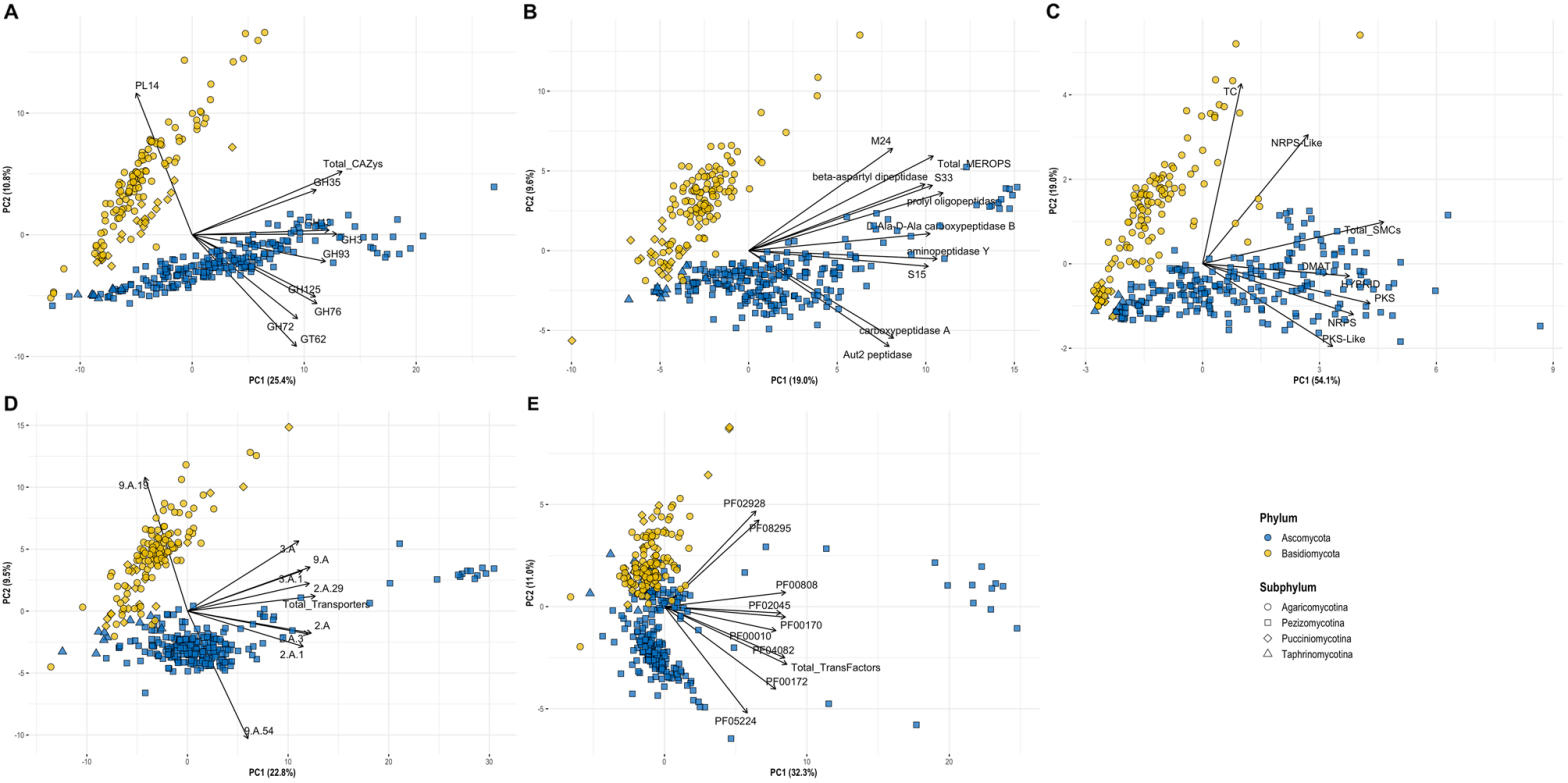
Principal component analysis (PCA) biplots of annotation data from (**A**) carbohydrate active enzymes, (**B**) peptidases, (**C**) secondary metabolite clusters, (**D**) transporters, and (**E**) transcription factors, showing the separation of the Ascomycota and Basidiomycota phyla on the first two principal components (PCs). The two phyla are differentiated by colour, and the four subphyla are differentiated by shape. The black arrows represent the loadings of the variables included in each PCA; only the top ten variables contributing to PC1 and PC2 are shown.

**Figure 3:**
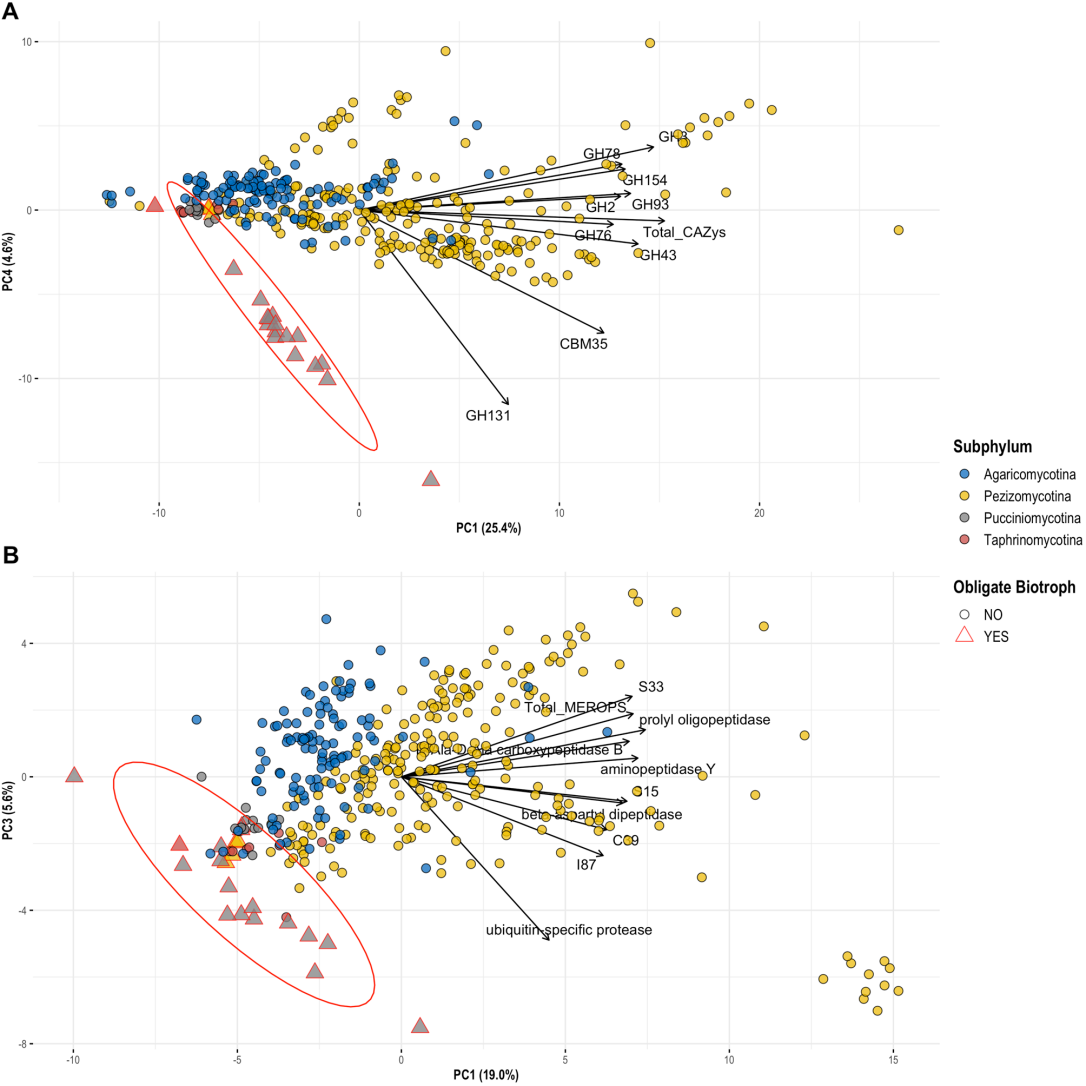
Principal component analysis (PCA) biplots for (**A**) carbohydrate active enzymes (CAZymes), and (**B**) peptidases, showing the clustering of obligate biotroph (OB) genomes. The four subphyla are differentiated by colour, and the obligate biotroph genomes are differentiated by shape. A statistical ellipse was drawn at a 95% confidence level around the OB genomes. The black arrows represent the loadings of the variables included in each PCA; only the top ten variables contributing to PC1 and PC4 (CAZyme PCA), or PC1 and PC3 (peptidase PCA), are shown.

Our fungal lifestyle database, FunLifeDB, comprising information on 533 fungal species (582 genomes; https://biosafe.cs.mcgill.ca/funlifedb/) was the source of lifestyle and trait data for the subset of 387 published genomes (from 355 species) we analysed (Suppl. Data S1, Fig. 1). We observed a strong phylogenetic signal in the genomic data. All PCAs using the genomic features showed a clear separation of the Ascomycota (Pezizomycotina and Taphrinomycotina) and Basidiomycota (Pucciniomycotina and Agaricomycotina), particularly on the first two principal components (Fig. 2). Still, the obligate biotrophs of both Ascomycota and Basidiomycota clustered together on the PCAs for CAZymes and peptidases, indicating common genomic features for this lifestyle (Fig. 3). While the phylogenetic signal for the obligate biotrophs was still evident in these PCAs, particularly for members of the order Pucciniales, there was also a clear similarity in the genomic profiles of species with this lifestyle regardless of their phylogenetic placement (Fig. 3). For all the other lifestyles and traits, including the plant pathogens, we did not find any clear patterns in the PCAs (data not shown).

Genomic patterns associated with fungal lifestyles and traits were revealed with the DendroNet machine learning algorithm, which showed that many lifestyles and traits could be predicted using both phylogenetic and genomic data. The inclusion of genomic features increased predictive performance over the parsimony models for all lifestyles and phytopathogenic traits we tested.

**Figure 4:**
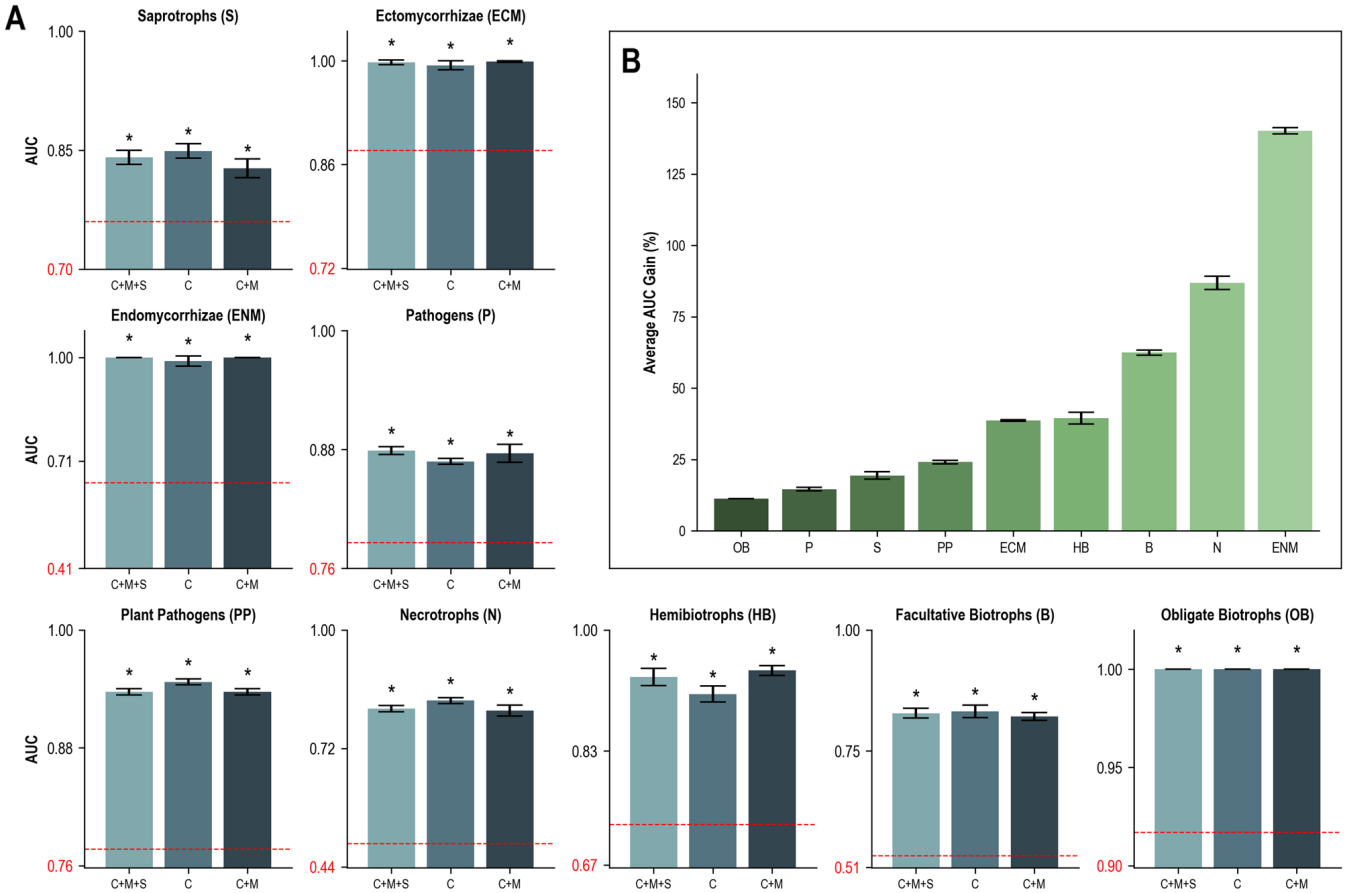
Area under the receiver operating characteristic curve (AUC) scores obtained with DendroNet for predicting nine lifestyles in fungi with the three top-performing genomic feature sets (C: CAZymes; M: MEROPS (peptidases); S: secondary metabolite clusters). Results from DendroNet’s lifestyle predictions for all 31 genomic feature sets are reported in Supplementary Data S2. (**A**) the average AUC score obtained for each lifestyle with only the phylogenetic data (parsimony model) is represented in red at the base of each graph (standard deviation of this value is represented with a dotted red line) and the average improvement of the model with the genomic feature(s) is represented with a blue column. The asterisk indicates that the AUC score from the genomic feature model was significantly (p < 0.05) greater than the AUC score from the parsimony model. (**B**) Percent gain ([[AUC parsimony–AUC genomic signal]/AUC parsimony] × 100) calculated for each lifestyle (average and standard deviation calculated from the three genomic feature sets).

The contribution of phylogeny to DendroNet’s lifestyle predictions varied greatly, with parsimony AUC scores ranging from 0.438 ± 0.057 for the necrotrophs to 0.899 ± 0.018 for the obligate biotrophs (Suppl. Data S2). The three genomic feature sets that produced the highest mean AUC scores for lifestyle predictions were the combination of CAZymes + MEROPS + SMCs (0.915 ± 0.076), the CAZymes alone (0.914 ± 0.071), and the combination of CAZymes + MEROPS (0.912 ± 0.081), respectively (Fig. 4, Suppl. Data S2). All three of these top-performing feature sets resulted in statistically significant (p < 0.05) increases in AUC scores over the parsimony models for every lifestyle tested (Suppl. Data S3). Prediction of the endomycorrhizal lifestyle improved the most from the addition of genomic features, with an average AUC increase of 0.582 across the top three feature sets (Fig. 4A), corresponding to a gain of 140% (Fig. 4B). Within the plant pathogenic lifestyles, DendroNet’s predictions improved the most for necrotrophs; the AUC scores increased by up to 0.395 (CAZyme feature set; Fig. 4A), with an average AUC gain of 87% over parsimony across the top three feature sets (Fig. 4B). The obligate biotroph lifestyle had the highest parsimony AUC of 0.899 ± 0.018 (Fig. 4A), resulting from a strong phylogenetic signal (there are only three orders within which obligate biotrophs are found), but increased to an AUC of 1.000 ± 0.000 (gain over the phylogeny signal of 11.2%) in all three of the top-performing genomic feature sets (Fig. 4B). After the obligate biotrophs, prediction scores for hemibiotrophs were the highest of the phytopathogenic lifestyles, with AUC scores of up to 0.943 ± 0.007 (CAZymes + MEROPS feature set), an improvement of 41.6% over the parsimony score (Fig. 4A and B). DendroNet also consistently predicted fungal pathogenicity (AUCs up to 0.879 ± 0.004 with CAZymes + MEROPS + SMCs) and plant pathogenicity (AUCs up to 0.947 ± 0.003 with CAZymes). However, phylogeny contributes a large proportion to this predictive capacity, with parsimony AUCs of 0.763 ± 0.023 and 0.758 ± 0.019 and gains over the phylogeny signal of 15.2% and 24.9% for the pathogen and plant pathogen lifestyles, respectively (Fig. 4A and B).

**Figure 5:**
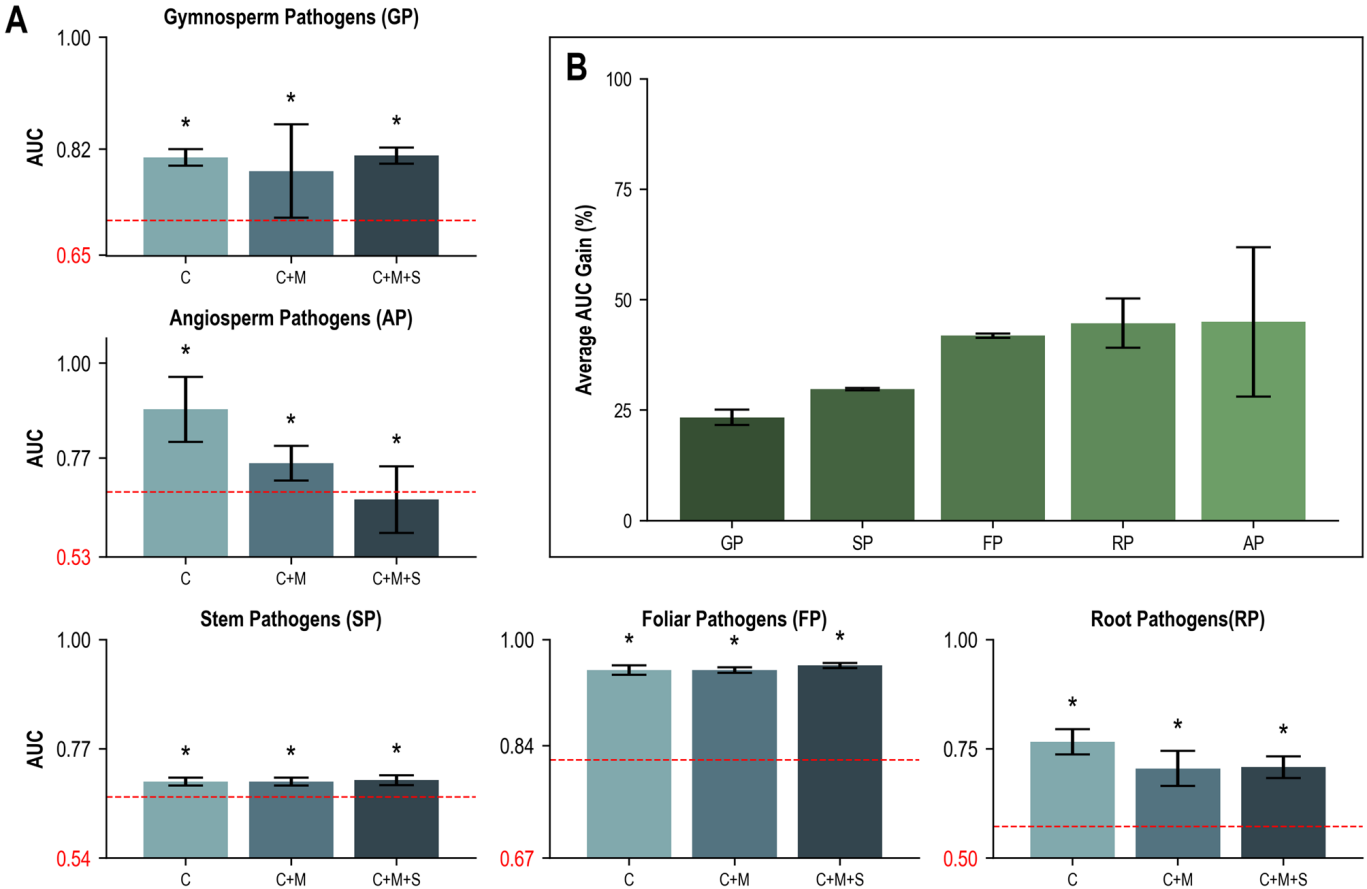
Area under the receiver operating characteristic curve (AUC) scores obtained with DendroNet for predicting five phytopathogenic traits in fungi with the three top-performing genomic feature sets (C: CAZymes; M: MEROPS (peptidases); S: secondary metabolite clusters). Results from DendroNet’s trait predictions for all 31 genomic feature sets are reported in Supplementary Data S2. (**A**) the average AUC score obtained for each trait with only the phylogenetic data (parsimony model) is represented in red at the base of each graph (standard deviation of this value is represented with a dotted red line) and the average improvement of the model with the genomic feature(s) is represented with a blue column. The asterisk indicates that the AUC score from the genomic feature model was significantly (p < 0.05) greater than the AUC score from the parsimony model. (**B**) Percent gain ([[AUC parsimony–AUC genomic signal]/AUC parsimony] × 100) calculated for each trait (average and standard deviation calculated from the three genomic feature sets).

The phylogenetic signals for the phytopathogenic traits tested (host type and tissues) ranged from parsimony AUCs of 0.502 ± 0.070 (root pathogens) to 0.674 ± 0.144 (foliar pathogens) (Suppl. Data S2). The inclusion of genomic data improved DendroNet’s predictions of all phytopathogenic traits, though the AUC gains were not as high as they were for the lifestyles (Fig. 5, Suppl. Data S2). The three genomic feature sets that produced the highest average AUC scores for predicting phytopathogenic traits were the CAZymes alone (0.823 ± 0.100), the combination of CAZymes + MEROPS (0.780 ± 0.103), and the combination of CAZymes + MEROPS + SMCs (0.770 ± 0.119), respectively. All three of these top-performing feature sets resulted in statistically significant (p < 0.05) increases in AUC scores over the parsimony models for every trait tested (Suppl. Data S3). DendroNet’s predictions of angiosperm pathogens improved the most over the parsimony model, though the variation in performance between the top three feature sets was more than for other traits (Fig. 5). Foliar pathogenicity was the best-predicted trait, with a mean AUC score of 0.956 ± 0.004 from the top three feature sets (Fig. 5A).

### Expansions and contractions of specific CAZyme, peptidase and secondary metabolite cluster gene families drive DendroNet’s predictions of phytopathogenic lifestyles and traits

The three top-performing genomic feature sets across phytopathogenic lifestyles and traits were CAZymes alone, CAZymes + MEROPS, and CAZymes + MEROPS + SMCs (Suppl. Data S2). Patterns in specific gene families arose as drivers for DendroNet’s predictions (Suppl. Data S4).

#### CAZymes

**Figure 6:**
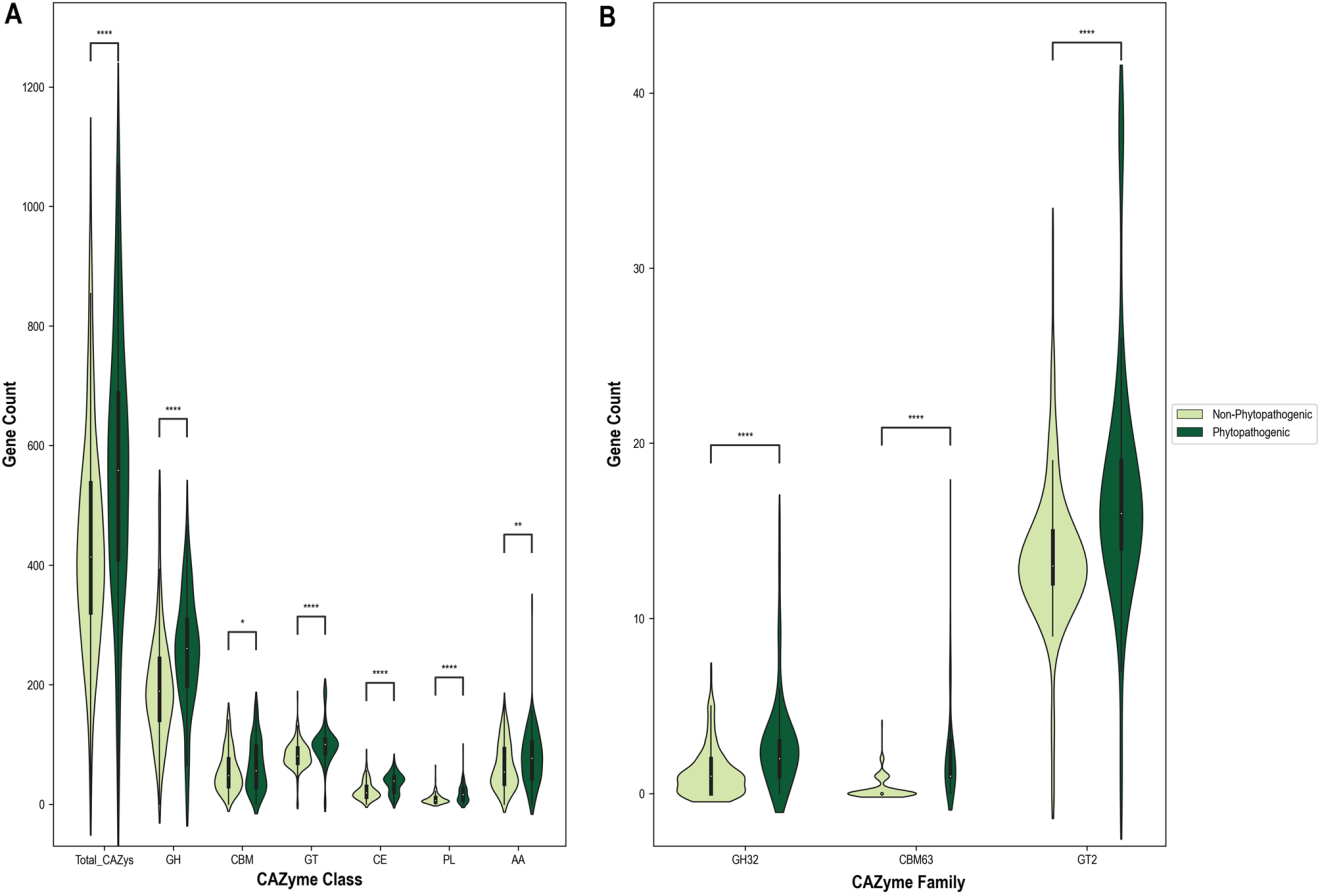
Violinplots of the gene counts from carbohydrate active enzyme (CAZyme) annotations revealed by DendroNet analyses to be expanded in phytopathogenic genomes relative to non-phytopathogenic genomes. (**A**) Gene counts of the six CAZyme classes and the total CAZymes (all CAZyme classes and families). (**B**) Gene counts of the three CAZyme families that were drivers for DendroNet’s predictions of fungal phytopathogenicity. The asterisks in both (**A**) and (**B**) indicate that the gene counts in phytopathogenic genomes were significantly greater than the gene counts in non-phytopathogenic genomes as per an independent *t* test (*p < 0.05, **p < 0.01, ***p < 0.001, ****p < 0.0001).

The total number of CAZymes in the genome and the number of genes from the glycoside hydrolase (GH), carbohydrate-binding module (CBM), and auxiliary activity (AA) CAZyme classes were increased in saprotrophs and plant pathogens, compared to the other lifestyles (Suppl. Fig. S1). The saprotrophic lifestyle was associated with a decrease of the carbohydrate esterases (CEs), polysaccharide lyases (PLs), and glycosyltransferases (GTs), while the phytopathogenic lifestyle was associated with an increase of all these gene classes (Fig. 6). Ectomycorrhizal genomes had decreased numbers of all CAZyme classes, including total CAZymes, whereas endomycorrhizal genomes had decreased numbers of AAs, CBMs, CEs, and PLs, but increased numbers of GHs, GTs, and total CAZymes. While none of the individual CAZyme families predicted plant pathogenicity better than using all of them together (AUC of 0.95; Fig. 4A), the best individual CAZyme predictor of plant pathogenicity was an increase of the GT class of genes (AUC of 0.91; Suppl. Data S4). Within the GT class, an increase of genes in the family GT2 was associated with plant pathogens (AUC of 0.85; Fig. 6, Suppl. Data S4), while a decrease of GT2 genes was the top individual predictor for saprotrophs (AUC of 0.83; Suppl. Data S4). The second-best predictor of plant pathogenicity was the number of CBM63 genes (AUC of 0.90; Suppl. Data S4), with an increased number in plant pathogens relative to non-plant pathogens (Fig. 6). Every phytopathogenic lifestyle except the biotrophs was associated with an increase in CBM63 genes, whereas saprotrophs and both mycorrhizal lifestyles were associated with a decrease in these genes (Suppl. Fig. S1, Suppl. Data S4). Another important predictor for plant pathogens was the number of GH32 genes (Fig. 6): almost every phytopathogenic lifestyle as well as endomycorrhizal fungi had increased GH32 genes, but biotrophs, saprotrophs, and ectomycorrhizal fungi showed decreased numbers (Suppl. Fig. S1, Suppl. Data S4, S5). An increase in GH32 genes was also associated with all phytopathogenic traits except for gymnosperm-infecting pathogens, which showed a decrease (Suppl. Data S4, S5). There were three lifestyles (biotrophs, hemibiotrophs, and ectomycorrhizal fungi) and three traits (angiosperm-, root- and stem-infecting pathogens) for which there were individual CAZyme families that provided better DendroNet predictions than when all CAZyme families were used together (Suppl. Data S4).

#### Peptidases

**Figure 7:**
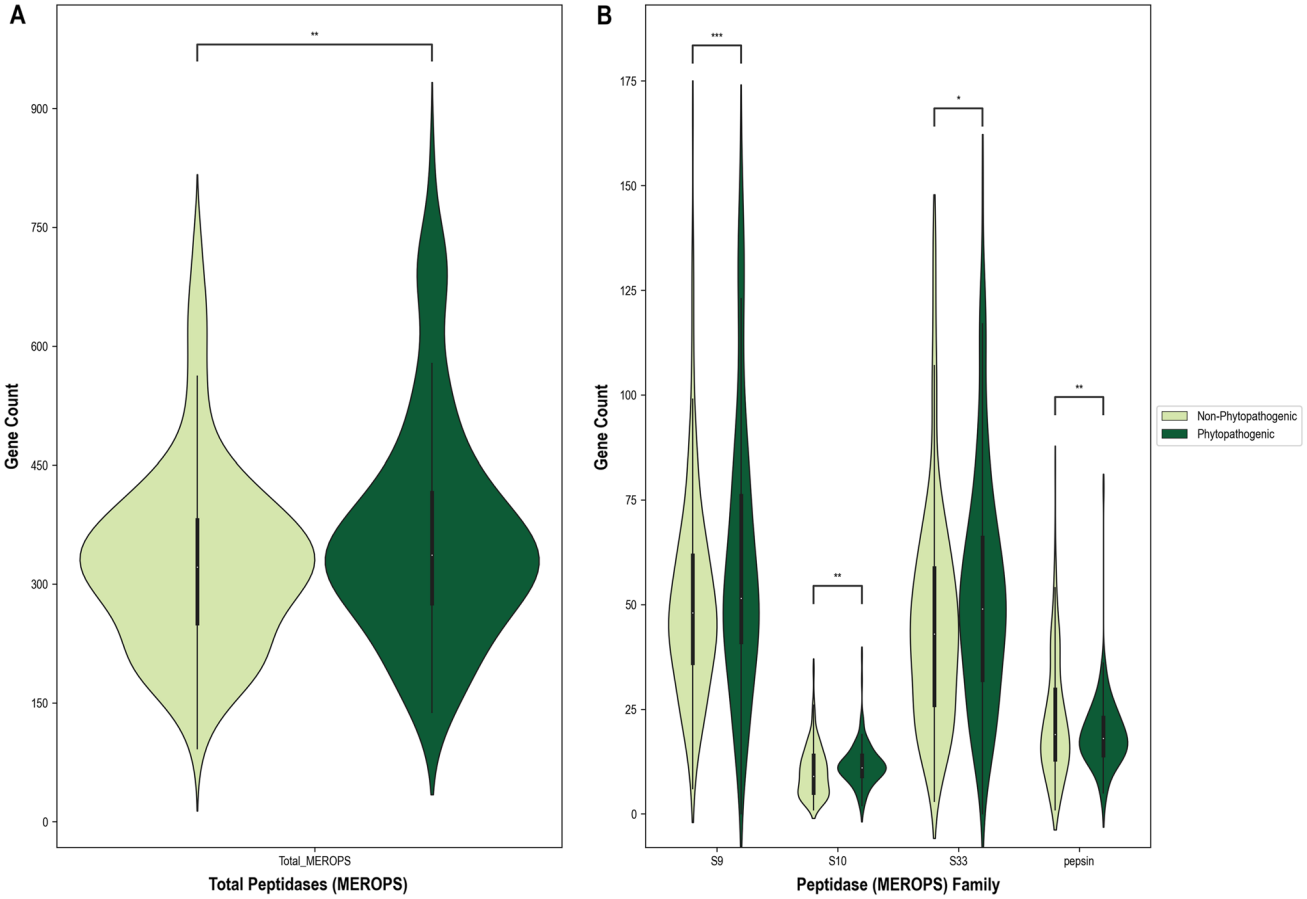
Violinplots of the gene counts from peptidase (MEROPS) annotations revealed by DendroNet analyses to be expanded or contracted in phytopathogenic genomes relative to non-phytopathogenic genomes. (**A**) Gene counts of the total peptidases (all clans and families). (**B**) Gene counts of the four peptidase families that were drivers for DendroNet’s predictions of fungal phytopathogenicity. The asterisks in both (**A**) and (**B**) indicate significance in gene counts as per an independent *t* test (*p < 0.05, **p < 0.01, ***p < 0.001, ****p < 0.0001). For the total peptidases (‘Total_MEROPS’), S9, S10, and S33 annotations, gene counts were significantly greater in phytopathogenic genomes relative to non-phytopathogenic genomes, whereas for pepsins, gene counts were significantly lower in phytopathogenic genomes relative to non-phytopathogenic genomes.

Several individual peptidase families produced better predictions than using all families combined for many of the lifestyles and traits (Suppl. Data S4). These peptidase families varied depending on the specific lifestyle or trait, but four recurring top predictor features were the prolyl oligopeptidase (S9), prolyl aminopeptidase (S33), and carboxypeptidase Y (S10) serine peptidase families as well as the pepsin A aspartic protease family (Suppl. Data S4). The genomes of plant pathogens exhibited increased numbers of S9, S10, and S33 genes, but decreased numbers of pepsin genes relative to non-phytopathogenic genomes (Fig. 7, Suppl. Fig. S2). An important predictor for all phytopathogenic lifestyles was the number of prolyl aminopeptidase (S33) genes; plant pathogen, necrotroph, hemibiotroph, and facultative biotroph genomes had increased S33 genes, while obligate biotroph genomes had decreased S33 genes (Suppl. Data S4). An increase in the total number of peptidases was the best individual predictor of plant pathogenicity (AUC of 0.88; Fig. 7, Suppl. Data S4) while the second-best predictor was an increase in genes from the S9 prolyl oligopeptidase family (AUC of 0.86; Fig. 7, Suppl. Data S4). The total number of peptidases was also an important predictor for all phytopathogenic lifestyles, with necrotrophs and hemibiotrophs exhibiting increased peptidases, and biotrophs (facultative and obligate) exhibiting decreased peptidases (Suppl. Fig. S2, Suppl. Data S4).

#### Secondary metabolite clusters

Only two lifestyles (ectomycorrhizal fungi and obligate biotrophs) and two traits (foliar and stem pathogens) had individual SMC features that improved prediction over the parsimony model (Suppl. Data S4). For all four of these groups, the total number of SMC genes was the strongest predictor, with ectomycorrhizal fungi, obligate biotrophs, and foliar pathogens having decreased total SMCs and stem pathogens having increased total SMCs (Suppl. Fig. S3, Suppl. Data S4). For ectomycorrhizal fungi, foliar pathogens, and stem pathogens, the fraction score of the total SMC feature was greater than one (Suppl. Data S4), indicating that the total number of SMC genes was a better predictor of these lifestyles than using all SMC families combined. The total number of SMC genes was also increased in the genomes of plant pathogens, including necrotrophs and hemibiotrophs, and endomycorrhizal fungi, but was decreased in the genomes of facultative biotrophs and saprotrophs (Suppl. Fig. S3, Suppl. Data S5).

## Discussion

Since Anton de Bary first discussed the concept of ‘nutritive adaptation’ in the late 1800s^36^, plant pathologists have been exploring and refining the definitions of trophic modes, or lifestyles, for fungal species^18,37–39^. Identifying the lifestyles of pathogenic fungi provides important information on how pathogens cause disease, how they spread through an ecosystem, and ultimately, how best to approach disease mitigation^12,18,40^. Our results support the hypothesis that there are genomic patterns, or signatures, associated with the lifestyles and ecological traits of fungal plant pathogens across phylogenetic groups and that these signatures can be used in a predictive capacity. While we observed a strong association between phylogeny and many of the lifestyles and traits, the inclusion of genomic data always improved the prediction performance of DendroNet, suggesting that there are genomic signatures beyond those shaped solely by phylogenetic relationships. Our study improves our understanding of the genomics underlying fungal phytopathogenic lifestyles and traits, while also highlighting some of the challenges and limitations of integrating large-scale genomic and lifestyle data into future biosurveillance practices.

The lack of lifestyle-associated patterns in our PCAs contrasts with recent results obtained by Hane et al.^20^, who used a PCA-based machine learning method (CATAStrophy) for lifestyle predictions in fungi and oomycetes. While Hane et al. did observe a strong phylogenetic signal in their PCAs, they also observed clusters of species with similar lifestyles that were phylogenetically unrelated^20^. The difference in the strength of the phylogenetic signal between the two studies could be an effect of sample size: 355 fungal species were used in our study *vs.* 158 in the CATAStrophy study^20^. As the number of species analyzed increases, the phylogenetic signal from the 452 million years of divergence between the Ascomycota and Basidiomycota^41,42^ might overwhelm any other genomic signals. The DendroNet machine learning approach allowed us to characterize the influence of phylogeny seen in our PCAs while exploring genomic signals outside phylogeny that are drivers of fungal phytopathogenic lifestyles and traits.

From a biosurveillance perspective, the most promising result is DendroNet’s ability to predict plant pathogenicity with AUC scores of up to 0.95. While the parsimony scores for plant pathogens indicate a strong phylogenetic signal, the increase of 25% in AUC with the inclusion of CAZyme data suggests there are specific genomic features contributing to plant pathogenicity in fungi. This finding validates recent results obtained with a subset of the MycoCosm database^21^, which demonstrated that within the Dothideomycetes, plant pathogens could be distinguished from saprobes with greater than 95% accuracy using machine learning on genomic data. Our analyses, performed on a larger number of species with a broader range of both taxonomic and lifestyle groups, confirm that not only can plant pathogens be distinguished from saprotrophs, but they also have a unique profile relative to other pathogens. Additionally, the high predictive performance of DendroNet with plant pathogen host type (angiosperms vs. gymnosperms), and particularly with foliar pathogens, indicates that there are also strong genomic signals associated with phytopathogenic traits. These findings related to plant pathogenic fungi have important implications for biosurveillance, specifically for predicting the lifestyles and traits of novel or unknown species. However, as with all machine learning algorithms, DendroNet’s performance is limited by the data that it was given for training. There is occasionally disagreement in the literature as to which lifestyle a fungal species exhibits^43^, and as fungal-plant interactions are studied more closely in the lab, previously unobserved lifestyles can be revealed^44^ and lifestyle assignments will thus shift. We addressed this lifestyle flexibility by assigning multiple lifestyles to species when supported by evidence from the literature. Additionally, when we encountered disagreement in the literature regarding the lifestyle assignment of a species, we chose the lifestyle for which the majority of studies had categorized a fungus. This inconsistency in the categorization of fungal lifestyles is a limitation of studies such as ours that aim to assign lifestyles and traits from the literature and highlights a significant challenge in this research moving forward. Our results provide strong support for the functionality of predictive genomics, but as the lifestyles and traits of more fungi are curated and updated, DendroNet should continue to be trained and tested to determine whether its predictive power extends to a broader range of taxonomic groups and lifestyles.

While we tested all possible combinations of the five genome annotations included in this study, using CAZyme data alone produced the best prediction results for most of the phytopathogenic lifestyles and traits, and CAZymes were also included in all three of the top-performing feature sets. This result is not surprising given that CAZymes are crucial for most fungal plant pathogens, allowing them to colonize their hosts by overcoming the barrier of the plant cuticle, remodeling the fungal cell wall to avoid recognition, and deconstructing the host cell wall^45–47^. Our finding that CAZymes are important predictors of fungal lifestyles is further supported by previous work demonstrating the predictive capacity of CAZyme content for filamentous phytopathogens^20^. Differences in the patterns we observed in non-phytopathogenic and phytopathogenic lifestyles further reflect the importance of CAZymes for plant pathogenic fungi, with the genomes of plant pathogens exhibiting expansions in many CAZyme classes compared to the genomes of non-phytopathogenic fungi (saprotrophs, endo- and ectomycorrhizal fungi). An increase in CAZyme content and activity for phytopathogenic fungi relative to saprotrophs has also been documented in previous studies^48–50^. While saprotrophs require CAZymes for the breakdown of dead plant tissues and nutrient assimilation, they do not require the extensive arsenal necessary for interacting with living plant tissues, nor are they subject to the diversifying selection that results from the co-evolutionary arms race between plants and their fungal pathogens^51–53^. Our results show that this difference in CAZyme content is also present between plant pathogens and mycorrhizal fungi, suggesting that the expansion of CAZymes in phytopathogenic species is driven by their antagonistic interactions with plants and is not simply a requirement for interacting with live plant tissues.

The expansion of glycosyltransferase (GT) genes, particularly in the GT2 family (chitin synthases), that we observed as an important predictor of plant pathogenicity contrasts previous findings that dothideomycete plant pathogens had a marked decrease in GTs in their genomes^34^. This discrepancy highlights the importance of increasing the availability of genome data for fungal phytopathogens from diverse taxa so that variations in genomic patterns can be observed. While GTs are well-known to be involved in fungal cell wall synthesis and have been proposed as targets for antifungal treatment of human pathogens^54^, their specific role in plant pathogens is still understudied. There is some evidence that GT2 orthologues may have been important in the evolution of fungal pathogens and likely play an important role in pathogenesis on plants^55^. While our results do show a contraction in GT2 genes in saprotrophs, we found an expansion of GT2s in both plant pathogenic and mycorrhizal genomes, suggesting that this CAZyme family is not solely important for pathogenesis, but rather involved somehow in plant-fungus interactions. The second most important predictor of plant pathogenicity was an expansion of genes in the CBM63 family, which are non-catalytic cellulose-binding modules appended to proteins with similarity to plant expansins^56^. Expansins are proteins that loosen plant cell walls and have been implicated in the virulence and plant-colonizing abilities of microbial phytopathogens^57–60^. CBM63-containing proteins were reported to play an important role during plant infection in *Botrytis cinerea*^61^ and *Fusarium oxysporum* f. sp. *pisi*^62^. Our finding that CBM63 genes are expanded in plant pathogens, but not in saprotrophs or mycorrhizal fungi, strongly suggests that plant cell wall loosening may be an important determinant in the evolution of plant pathogenicity.

The expansion of GH32 genes (invertases) that we observed in plant pathogens in general, and more specifically for necrotrophs as well as foliar, root, and angiosperm pathogens contrasts with the contraction of these genes in biotrophic species. GH32 enzymes are invertases that hydrolyze sucrose to glucose and fructose, and evidence suggests that they are used by fungal pathogens to obtain carbon from their plant hosts^63,64^. GH32 was expressed during the pathogenic activities of biotrophic fungi^64–66^, and an expansion of these genes has been demonstrated in plant pathogen genomes relative to saprotrophic and mycorrhizal species^63,67^, suggesting that GH32 genes could be important in the evolutionary history of plant pathogenicity. In fact, the number of GH32 gene copies has been previously proposed as a predictor of the ecological strategies of fungi^63,67^, lending further support to our findings that this CAZyme subfamily is a major predictor of many plant pathogenic lifestyles.

We also observed that patterns in specific peptidase families, particularly serine proteases, were associated with plant pathogenicity. Serine proteases have been demonstrated by numerous studies to be important in fungal phytopathogenicity^68^, and an expansion in the S9 (prolyl oligopeptidase), S10 (carboxypeptidase Y), and S33 (prolyl aminopeptidase) families has been reported for plant-associated fungi^69^. Prolyl aminopeptidases, a family that was expanded in every phytopathogenic lifestyle except obligate biotrophs, are enzymes involved in the cleavage of N-terminal proline residues from peptides^70^. There has been evidence that proteins with proline-rich N-terminal domains are involved in the maintenance of biotrophy^39,71^, so it could be that obligate biotrophs require less of the enzymes that would cleave such proline-rich domains. A contraction of pepsins (MEROPS clan AA, family A1) was an important predictor of plant pathogens in general as well as necrotrophs, hemibiotrophs, and foliar pathogens. Family A1 are aspartic proteases, which are thought to play a role in the virulence of plant pathogenic fungi^72,73^, so it is somewhat unexpected that we observed a contraction in this family for plant pathogenic lifestyles. Future research could expand analyses within family A1 to determine the specific subfamilies driving these contractions.

Genome-based predictive approaches have important implications for the biosurveillance of invasive plant pathogens as there has been a call for more genomics-centered biosecurity strategies, especially for forest invasive alien species (FIAS)^8–10,12^. Here we demonstrate that predictive genomics is a promising tool that could be harnessed for biosurveillance of fungal phytopathogens. In addition to its predictive performance, our machine learning approach uncovered genomic patterns associated with specific phytopathogenic lifestyles and traits, elucidating gene families that are potentially important in the evolution of plant pathogens. These results indicate that while evolutionary differentiation is undoubtedly a major driving force for fungal lifestyles and traits, there are clearly other selective pressures influencing the genomic architecture of phytopathogenic fungi. This finding highlights the importance of moving away from solely taxonomy-focused biosurveillance towards more genomics-based strategies. Our approach used only a small group of gene families with readily available annotations; future research could be expanded to use whole genome data, including genes known to be important in fungal phytopathogenicity, such as effector proteins^74,75^. Additional phytopathogenic traits, such as host range (e.g. broad vs narrow, monocots vs dicots), should also be tested. The use of genomic patterns for prediction of fungal lifestyles is complex. Future genomic biosurveillance may require a tailored approach that depends on the specific group of pathogens being monitored. As more genomes become publicly available, we will be able to more robustly test the capacity of tools like DendroNet to predict fungal phytopathogenic lifestyles and traits, as well as assess the integration of these predictive tools into future FIAS biosurveillance pipelines.

## Methods

### Fungal lifestyle database

We created a lifestyle database for 533 fungal species (582 genomes) with data available from the Joint Genome Institute’s (JGI) MycoCosm Fungal Portal^25^. The species in our database span two ascomycete subphyla (Pezizomycotina, Taphrinomycotina) and two basidiomycete subphyla (Pucciniomycotina, Agaricomycotina). Each species was given taxonomic labels for phylum, class and order using the fungal nomenclature of Index Fungorum^76^. To categorise each of the species in our database into its respective lifestyle(s) and identify important ecological traits, we used both the information and references from MycoCosm as well as information found in an independent literature search. We used 1014 peer-reviewed publications in assigning lifestyles and traits to fungi in the database.

In total, we assembled a list of 24 different lifestyles to which species could be assigned (Table S1), including four lifestyles exhibited by important phytopathogens (biotroph, obligate biotroph, necrotroph, hemibiotroph) for both managed and natural plant systems. Given the subjectivity in the literature as well as the possibility that one species can exhibit multiple trophic strategies during its life cycle^18,44^, species were often assigned more than one lifestyle. In cases where there was disagreement in the literature as to the lifestyle a species exhibits, we chose the lifestyle on which the majority of published studies agreed. If a species could not be definitively categorized from the literature, it was labelled as “Unknown”. For the pathogenic fungi we included ten additional ecological traits relevant to pathogenicity (Table S2). For plant pathogenic species, host type (gymnosperms, angiosperms) and targeted tissues (stem, leaves, roots) were assigned and are hereby referred to as “phytopathogenic traits”. These phytopathogenic traits were determined only from environmental studies; experimental studies conducted in controlled conditions (e.g. artificial inoculations) were not used.

### Genome analyses

We used both principal component analysis (PCA) and machine learning to perform genomic comparisons amongst a subset of species from the database with available data. For machine learning, we used DendroNet, a phylogeny-aware method of training machine-learning models that incorporates both phylogenetic and genomic data^77^; this allowed us to separate the signals from phylogeny and gene content and determine which genomic features were associated with specific lifestyles and traits.

### Genome annotations

The genomic data for each MycoCosm species can only be used if there is an associated genome reference, or with explicit permission from the principal investigator (PI). Therefore, we downloaded the genome annotation data (gene counts) available from MycoCosm for a subset of 355 fungal species (387 genomes) from our lifestyle database consisting of 362 published and 25 unpublished genomes (used with PI permission). We obtained data from the five annotation groups available from MycoCosm: secondary metabolite clusters (SMCs–7 clusters), carbohydrate-active enzymes (CAZymes–220 families, subfamilies not included), peptidases (MEROPS–144 clans/families), membrane transport proteins (transporters–522 families), and transcription factors (transfactors–65 families). The gene counts from each of these five groups were then aligned to their respective species in the lifestyle database for comparative genomic analyses. While some of the genomes from MycoCosm were sequenced externally (see Data S1 for original genome reference papers), all the functional annotations were generated by the JGI Annotation Pipeline^25,78^, except for CAZymes, which were annotated by the Carbohydrate-Active enZYmes database (http://www.cazy.org/)^79^ in collaboration with the JGI. 

### Phytopathogenic lifestyles and traits

We performed the genomic analyses on the gene count data for all 387 genomes, but we used only a subset of lifestyles relevant to plant pathogenicity for labelling: pathogenic (includes animal, fungus, and plant pathogens), plant pathogenic (plant pathogens only), biotrophs (B), obligate biotrophs (OB), hemibiotrophs (HB), and necrotrophs (N). We also included saprotrophs (S) as a non-pathogenic lifestyle comparison, as well as ectomycorrhizal (ECM) and endomycorrhizal (ENM) species to compare plant-interacting, but non-pathogenic, fungi to phytopathogenic fungi. The ENM category comprised both ericoid mycorrhizal species and ectendomycorrhizal species. The phytopathogenic traits (gymnosperm, angiosperm, stem, foliar, and root pathogens) were also included as labels for the analyses.

### Principal component analyses

PCAs were performed using R software (ver 3.5.1)^80^. Pre-processing of the data for each PCA was performed as follows: all species rows with NA values (no gene annotation) were removed, variables with zero variance and near zero variance were removed from the data using the function nearZeroVar from the caret R package^81^, and the data were scaled to unit variance and centered. The function PCA was used from the R package factoextra^82^. We performed PCAs separately for each genome component (i.e. CAZymes, MEROPS, SMCs, transporters, and transfactors) to avoid excessive noise observed for joined data analyses^20^.

### DendroNet machine learning

#### Dataset preprocessing

Membership in each of the lifestyles and phytopathogenic traits described above was considered as a separate binary-classification problem for the machine learning analyses, and the corresponding gene counts from each annotation group were used as features. For each of the target lifestyle/trait classes, we used a total of 31 genomic feature sets to train a predictive model: each of the five annotations (CAZymes, MEROPS, SMCs, transporters, and transfactors) as individual genomic feature sets, and every possible combination of the annotations (26 possible combinations). We performed the machine learning analyses for the phytopathogenic traits only on species belonging to the plant pathogen class (subset of 138 species).

#### DendroNet architecture

DendroNet models have two components. The first component is a base model architecture, used to make predictions given a set of input data and a target output. The base model architecture used in this study was a logistic regression classifier. The second component is a neural network with the same topology as the phylogenetic tree that relates all the samples in the dataset. This neural network is used to determine the optimal weights to be used for the base classifier’s predictions at each location in the phylogenetic tree. Regularization is used to encourage the use of similar weights in species that are closely related. For this study, we retrieved the Dikarya (Ascomycota + Basidiomycota) phylogenetic tree from MycoCosm^25^, which was pruned to the species being analysed prior to input into DendroNet models. A pruned version of this tree with the 387 fungal genomes we analysed is included in Supplemental Data S6 in Newick format. To make the tree, proteins from all 387 genomes were clustered using MMseqs2^83^ (Version: 0188988235c6f1a8e90f327827c73f981db8a19a). Orthologous proteins were identified from the clusters allowing for paralogs and up to 100 missing genomes per cluster. When paralogs were present, only one copy from each genome was retained for alignment. Proteins from 2580 selected clusters were aligned using MAFFT^84^ (v7.123b). Divergent regions and poorly aligned positions were cleaned using Gblocks^85^ with options ‘− t = p–e = .gb − b4 = 5 − b5 = h’. The resulting cleaned alignments were concatenated and used for tree building with FastTree^86^.

#### Model training

We trained a separate DendroNet model for each lifestyle/trait classification task. Each model was trained for 1000 epochs using a learning rate of 0.001 and the Adam optimizer^87^. Regularization was applied to the L1 norm of the adjustments in weights made by the neural network, scaled by a factor of 1.0, via the process described previously^77^.

#### Model evaluation

We evaluated DendroNet model performance using the area under the receiver operating characteristic curve (AUC) and the results are reported on a ninefold cross-validation split. The AUC value indicates the ability of the model to distinguish between members and non-members of a given class. AUC scores range in value from 0.0 (model has no ability to distinguish between classes) to 1.0 (model’s predictions perfectly separate the classes). Machine learning models producing AUC scores less than 0.6 are considered inappropriate for a classification task while those above 0.7 are considered reasonable, and those producing scores above 0.8 are considered strong^88,89^.

#### Feature importance evaluation

We investigated the predictive power of each genomic feature set towards the lifestyle and trait classes. To separate the significance of a genomic feature set from the phylogenetic signal, we used the following process: first, the baseline predictive power of phylogeny was established for each target lifestyle and trait. This was done by training a DendroNet model that used only a bias term as a feature value, which produced predictions that were made using solely the phylogenetic placement of each species in the dataset, similar to a maximum parsimony tree. We therefore refer to this baseline phylogenetic prediction as the parsimony model. Next, for each genomic feature set, we trained a DendroNet model using both that feature set and a bias term, allowing for the use of phylogenetic placement information. We then compared the performance of this feature + parsimony model to the parsimony model alone, with an improvement in DendroNet’s performance indicating that the genomic feature set conveys information about the target lifestyle or trait beyond the phylogenetic signal. We also trained DendroNet models using each individual feature within a genomic feature set (e.g., GH5 family in CAZymes) and analysed the performance of these models relative to the model using all features of the same set (e.g. all CAZyme families) and relative to the parsimony model. If an individual feature improved DendroNet’s performance over the parsimony model for a given lifestyle or trait, we documented its corresponding AUC score increase over the parsimony model (reported as a ‘raw’ score) as well as the fraction of the total AUC improvement relative to the AUC score from the whole genomic feature set (reported as a ‘fraction’ score). Additionally, we recorded the correlation direction of the features for each lifestyle and trait (i.e. whether an increase or decrease in genes from each annotation group was associated with a lifestyle or trait).

#### Statistical analyses

Prior to performing statistical analyses, we determined the distributions of the relevant data with Shapiro–Wilk normality tests. To compare DendroNet’s performance across all 31 genomic feature sets, we performed a non-parametric analysis of variance with repeated measures (Friedman test) on the AUC scores for the lifestyles and traits (separate analyses) followed by a Nemenyi test for post-hoc analysis. To determine whether the feature + parsimony models resulted in a significantly increased AUC score compared to the respective parsimony models, we performed paired, one-tailed tests: either a paired *t*-test or a Wilcoxon signed-rank test depending on whether the paired differences followed a normal or non-normal distribution. To compare the gene counts from the top CAZyme and peptidase predictors for plant pathogenicity, we performed independent *t* tests on the gene counts from phytopathogenic genomes (‘Plant Pathogen’ = YES in FunLifeDB) relative to non-phytopathogenic genomes (‘Plant Pathogen’ = NO in FunLifeDB).

### Supplementary Information

Supplementary Information 1.

Supplementary Information 2.

Supplementary Information 3.

Supplementary Information 4.

Supplementary Information 5.

Supplementary Information 6.

Supplementary Information 7.

## Data Availability

The genomic datasets analysed in this study are available online from the Joint Genome Institute’s (JGI) MycoCosm Portal (https://mycocosm.jgi.doe.gov/mycocosm/home). All data generated during this study are included in this article and its Supplementary Information files.
