# Supplementary material for: Large-scale genomic analyses with machine learning uncover predictive patterns associated with fungal phytopathogenic lifestyles and traits
Source: Sci Rep. 2023 Oct 11;13:17203. doi: 10.1038/s41598-023-44005-w (PMC10567782; doi:10.1038/s41598-023-44005-w)
Supplement: Supplementary file 7 — Supplementary Information 7. [file 41598_2023_44005_MOESM7_ESM.docx]

Large-scale genomic analyses with machine learning uncover predictive patterns associated with fungal phytopathogenic lifestyles and traits – Supplementary Information

# Supplementary Results

## Results of statistical analyses run on DendroNet AUCs

DendroNet’s ability to predict fungal lifestyles varied greatly depending on the lifestyle and genomic features included in the model, with a range in AUC scores from 0.204 ± 0.185 (endomycorrhizal fungi using SMCs+transporters+transfactors feature set) to 1.000 ± 0.000 (obligate biotrophs in 26 feature sets) (Data S2). Statistical analysis with a Friedman test on the lifestyle prediction results showed a significant difference in DendroNet’s AUC scores depending on the genomic feature set used (χ² = 128.361, *p* = 3.932e-14). However, post-hoc analysis with a Nemenyi test revealed that these significant differences arose only between a few of the best- and worst-performing feature sets; the differences in DendroNet results between most of the 31 genomic feature sets were not statistically significant (Data S3). Two consistent patterns emerged from the Nemenyi test: the first was that the SMCs alone produced significantly lower AUC scores from DendroNet when compared to the top eight feature sets, and the second was that these top eight feature sets all included the CAZyme annotation data (Data S3).

DendroNet’s performance in predicting fungal phytopathogenic traits also varied greatly, with AUC scores ranging from 0.294 ± 0.015 (gymnosperm pathogens using SMCs+transfactors feature set) to 0.961 ± 0.004 (foliar pathogens using the CAZymes+MEROPS+SMCs feature set) (Data S4). As with the lifestyles, statistical analysis with a Friedman test on the trait predictions showed a significant difference in AUC scores depending on the genomic feature set used (χ² = 59.584, *p* = 0.001), though the Nemenyi post-hoc test showed that these differences arose only between the poorest feature set (SMCs alone) and the top three feature sets (Data S3).

# Supplementary Tables

**Table S1.** The list of 24 lifestyles to which fungal species were assigned in FunLifeDB based on a literature search, with the corresponding definitions. The lifestyles that were used for analyses in the current studies are highlighted in yellow.

| **Lifestyles (Abbreviation)** | **Definition** |
| --- | --- |
| Acidophile (A) | Fungi that can survive and grow optimally in highly acidic (pH < 3-4) environments. |
| Alkaliphilic (AL) | Fungi that can survive and grow optimally in highly alkaline (pH > 9) environments. |
| Biotroph (B) | Pathogenic fungi that grow, and often reproduce, within living host tissues. |
| Black Yeast (BY) | Fungi with highly melanized cell walls that can tolerate a wide range of extreme environments (polyextremotolerant). |
| Brown Rot (BR) | Wood-decaying fungi in the Basidiomycota that selectively degrade wood carbohydrates, but not lignin. |
| Coprophilous (C) | Saprobic fungi that grow and assimilate nutrients from animal dung. |
| Ectomycorrhizae (ECM) | Fungi that form a symbiotic association with plants by growing intercellularly in the roots. |
| Endolichenic (EL) | Fungi that grow asymptomatically within lichen thalli, akin to endophytes, but are not partners in the stable lichen symbiosis. |
| Endomycorrhizae (ENM) | Fungi that form a symbiotic association with plants by growing both inter- and intracellularly in the roots. |
| Endophyte (E) | Fungi that grow within plant tissues but are non-pathogenic. |
| Epiphyte (EP) | Fungi that grow outside or on plant tissues but are non-pathogenic. |
| Hemibiotroph (HB) | Pathogenic fungi that initially act as biotrophs within their plant host and then switch to a necrotrophic lifestyle. |
| Lichenicolous (L) | Fungi that grow obligately on lichens – can be pathogenic or saprotrophic. |
| Lichenized (LF) | Fungi that form a mutualistic symbiosis with algae as part of a lichen thallus. |
| Mycoparasite (MP) | Fungi that act as parasites of other fungi. |
| Necrotroph (N) | Pathogenic fungi that secrete toxins to kill their plant host and subsequently assimilate nutrients from the dead tissues. |
| Obligate Biotroph (OB) | Pathogenic fungi that require living host tissues to grow and reproduce – cannot survive outside their host. |
| Opportunistic/Latent Pathogen (O) | Fungi that only act as pathogens under certain environmental conditions, or towards hosts that have already been weakened by other biotic or abiotic agents. |
| Orchid Mycorrhizal (ORM) | Fungi that form mycorrhizal associations with orchid species. |
| Parasitic (P) | Fungi that grow in or on a living host; the fungus benefits from the association but there is a fitness cost for the host. Parasitic associations can range from weak to highly aggressive. |
| Pyrophilous (PY) | Fungi that produce fruiting bodies in post-fire environments. |
| Saprotroph (S) | Fungi that grow and feed on dead and decaying plant matter. |
| Thermophile (T) | Fungi that can survive and grow optimally in extremely high temperatures. |
| White Rot (WR) | Wood-decaying fungi in the Basidiomycota that degrade all wood components, including lignin. |

**Table S2.** The ecological traits assigned to pathogenic fungi in FunLifeDB; host type was assigned to all pathogens whereas only plant pathogens received additional assignments for plant host information and infected/inoculated tissues.

| **Ecological Traits** | |
| --- | --- |
| Host Type | Plant  Fungus  Vertebrate  Invertebrate |
| Plant Host Information | Gymnosperms  Angiosperms |
| Infected/Diseased Tissues | Main Stem  Leaves  Roots |

**Table S3.** The balance of genomes across pathogenic groups as well as the tested lifestyles and traits in both the original FunLifeDB and the reduced dataset of published genomes used for analyses.

|  | **Number of Genomes in Original Database** | **Number of Genomes in Reduced Database** |
| --- | --- | --- |
| Pathogens | 251 | 177 |
| Non-pathogens | 265 | 177 |
| Pathogenicity unknown | 66 | 33 |
| Plant pathogens | 196 | 138 |
| Saprotrophs | 369 | 241 |
| Ectomycorrhizae | 36 | 32 |
| Endomycorrhizae | 7 | 7 |
| Necrotrophs | 80 | 56 |
| Hemibiotrophs | 78 | 52 |
| Facultative biotrophs | 43 | 37 |
| Obligate biotrophs | 24 | 21 |
| Gymnosperm pathogens | 34 | 24 |
| Angiosperm pathogens | 181 | 128 |
| Stem pathogens | 131 | 99 |
| Foliar pathogens | 134 | 103 |
| Root pathogens | 64 | 40 |
| **Total species** | **533** | **355** |
| **Total genomes** | **582** | **387**  (362 published, 8 genomes from R.H. as PI, 17 genomes from F.M. as PI) |

#

# Supplementary Figures

**Figure S1.** Clustered heatmap of the CAZyme enzyme classes associated with DendroNet’s predictions of phytopathogenic lifestyles and traits. Also included is the ‘Total_CAZys’ feature, which is the total number of CAZyme genes annotated in each genome regardless of enzyme class. The values in the heatmap are the correlation directions for each CAZyme class input into DendroNet as a machine learning feature, which indicate whether an increase (positive correlation, red) or decrease (negative correlation, blue) in the number of genes in a CAZyme class was associated with a lifestyle or trait. Abbreviations: S, saprotrophs; ECM, ectomycorrhizal; ENM: endomycorrhizal; P, pathogens; PP, plant pathogens; N, necrotrophs; HB, hemibiotrophs; B, biotrophs; OB, obligate biotrophs; AP, angiosperm pathogens; GP, gymnosperm pathogens; FP, foliar pathogens; SP, stem pathogens; RP, root pathogens.


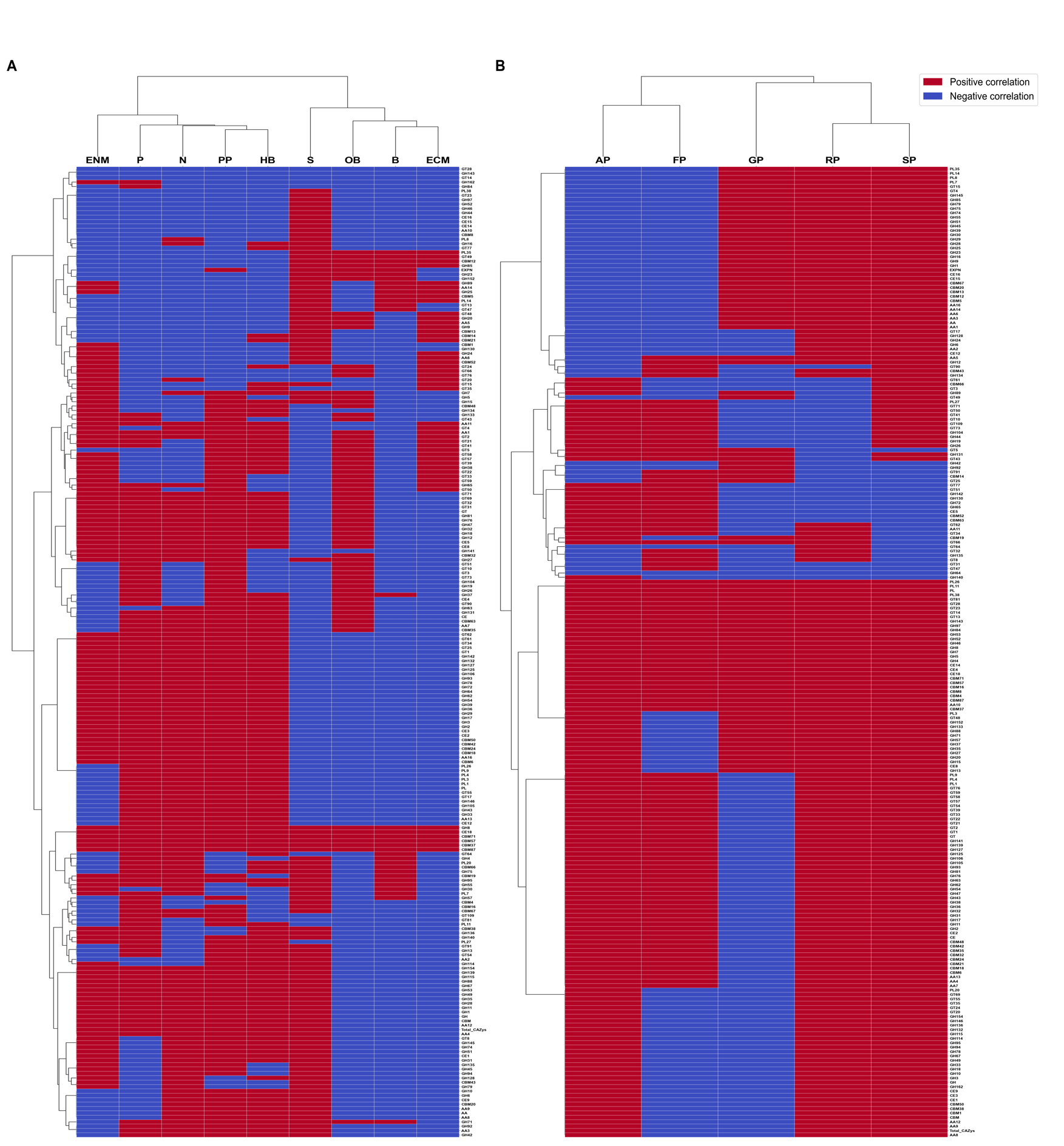


**Figure S2.**  Clustered heatmap of the peptidase (MEROPS) clans and families associated with DendroNet’s predictions of phytopathogenic lifestyles and traits. Also included is the ‘Total_MEROPS’ feature, which is the total number of peptidase genes annotated in each genome regardless of clan or family. The values in the heatmap are the correlation directions for each MEROPS clan/family input into DendroNet as a machine learning feature, which indicate whether an increase (positive correlation, red) or decrease (negative correlation, blue) in the number of genes in a group was associated with a lifestyle or trait. Abbreviations: S, saprotrophs; ECM, ectomycorrhizal; ENM: endomycorrhizal; P, pathogens; PP, plant pathogens; N, necrotrophs; HB, hemibiotrophs; B, biotrophs; OB, obligate biotrophs; AP, angiosperm pathogens; GP, gymnosperm pathogens; FP, foliar pathogens; SP, stem pathogens; RP, root pathogens.


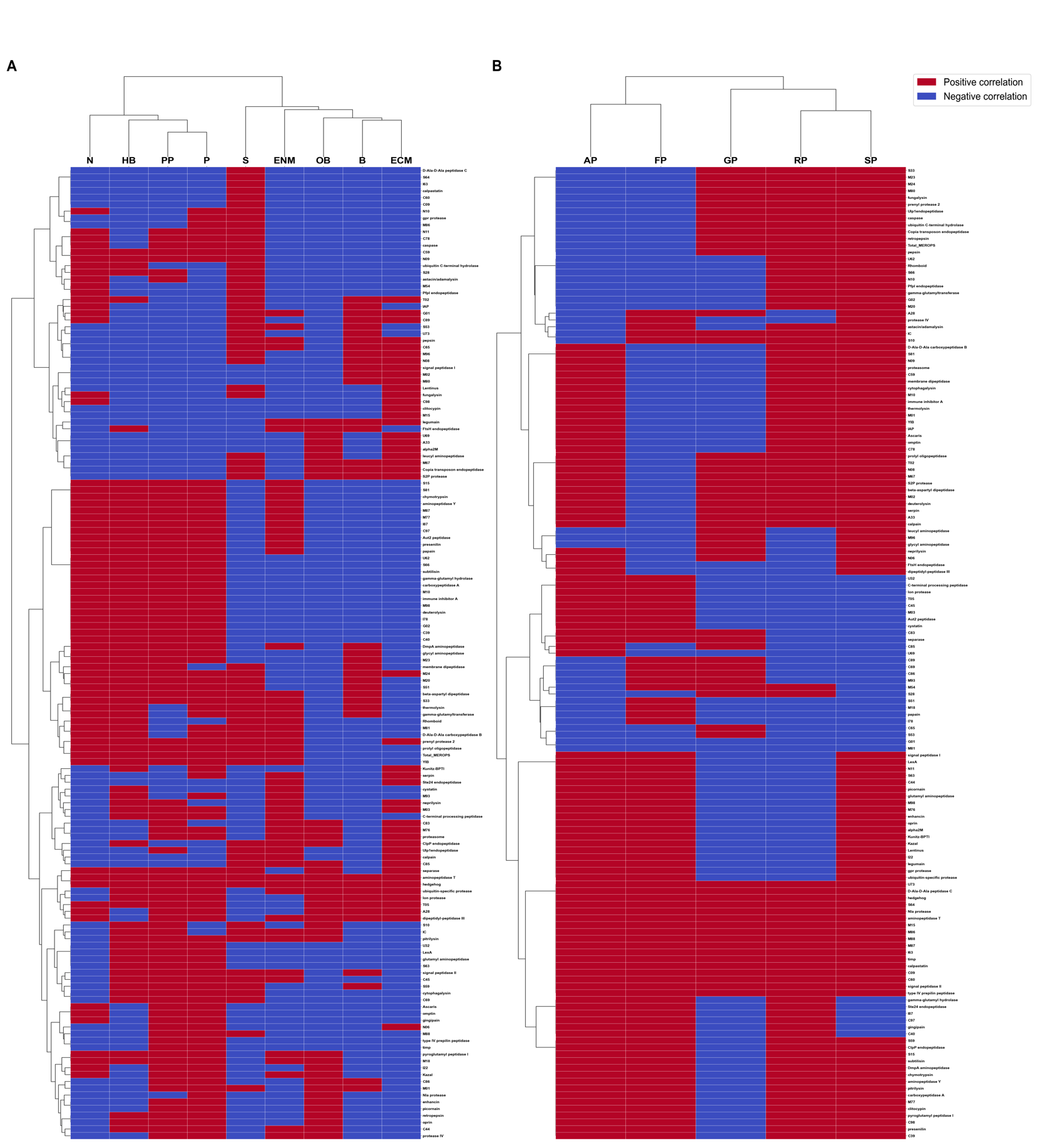


**Figure S3.** Clustered heatmap of the secondary metabolite cluster (SMC) classes associated with DendroNet’s predictions of phytopathogenic lifestyles and traits. Also included is the ‘Total_SMCs’ feature, which is the total number of SMC genes annotated in each genome regardless of class. The values in the heatmap are the correlation directions for each SMC class input into DendroNet as a machine learning feature, which indicate whether an increase (positive correlation, red) or decrease (negative correlation, blue) in the number of genes in a class was associated with a lifestyle or trait. Abbreviations: S, saprotrophs; ECM, ectomycorrhizal; ENM: endomycorrhizal; P, pathogens; PP, plant pathogens; N, necrotrophs; HB, hemibiotrophs; B, biotrophs; OB, obligate biotrophs; AP, angiosperm pathogens; GP, gymnosperm pathogens; FP, foliar pathogens; SP, stem pathogens; RP, root pathogens.

**
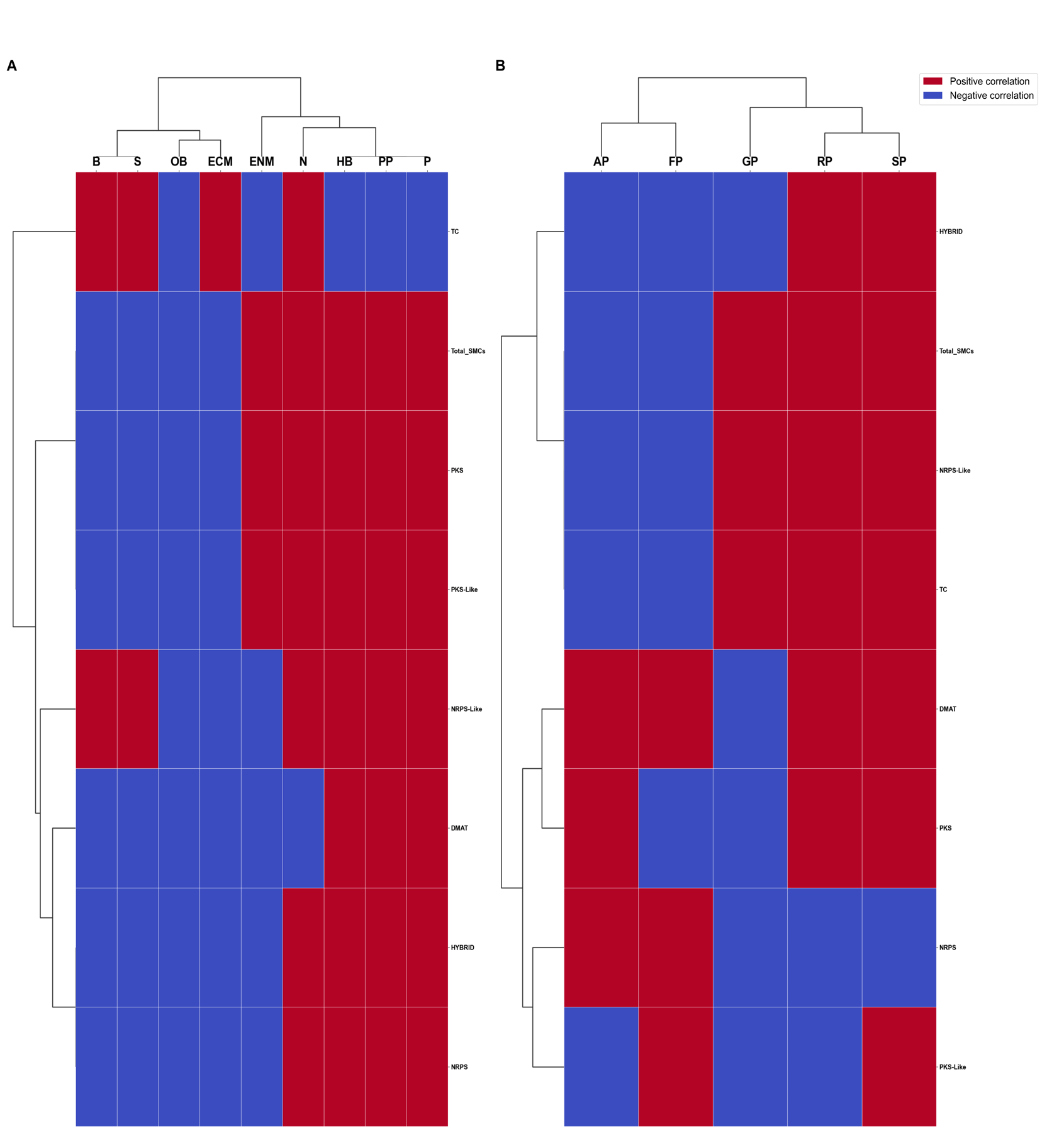
**

**Figure S4.** Clustered heatmap of the transporter superfamilies and families associated with DendroNet’s predictions of phytopathogenic lifestyles and traits. Also included is the ‘Total_Transporters’ feature, which is the total number of SMC genes annotated in each genome regardless of family. The values in the heatmap are the correlation directions for each transporter family input into DendroNet as a machine learning feature, which indicate whether an increase (positive correlation, red) or decrease (negative correlation, blue) in the number of genes in a family was associated with a lifestyle or trait. Abbreviations: S, saprotrophs; ECM, ectomycorrhizal; ENM: endomycorrhizal; P, pathogens; PP, plant pathogens; N, necrotrophs; HB, hemibiotrophs; B, biotrophs; OB, obligate biotrophs; AP, angiosperm pathogens; GP, gymnosperm pathogens; FP, foliar pathogens; SP, stem pathogens; RP, root pathogens.

**
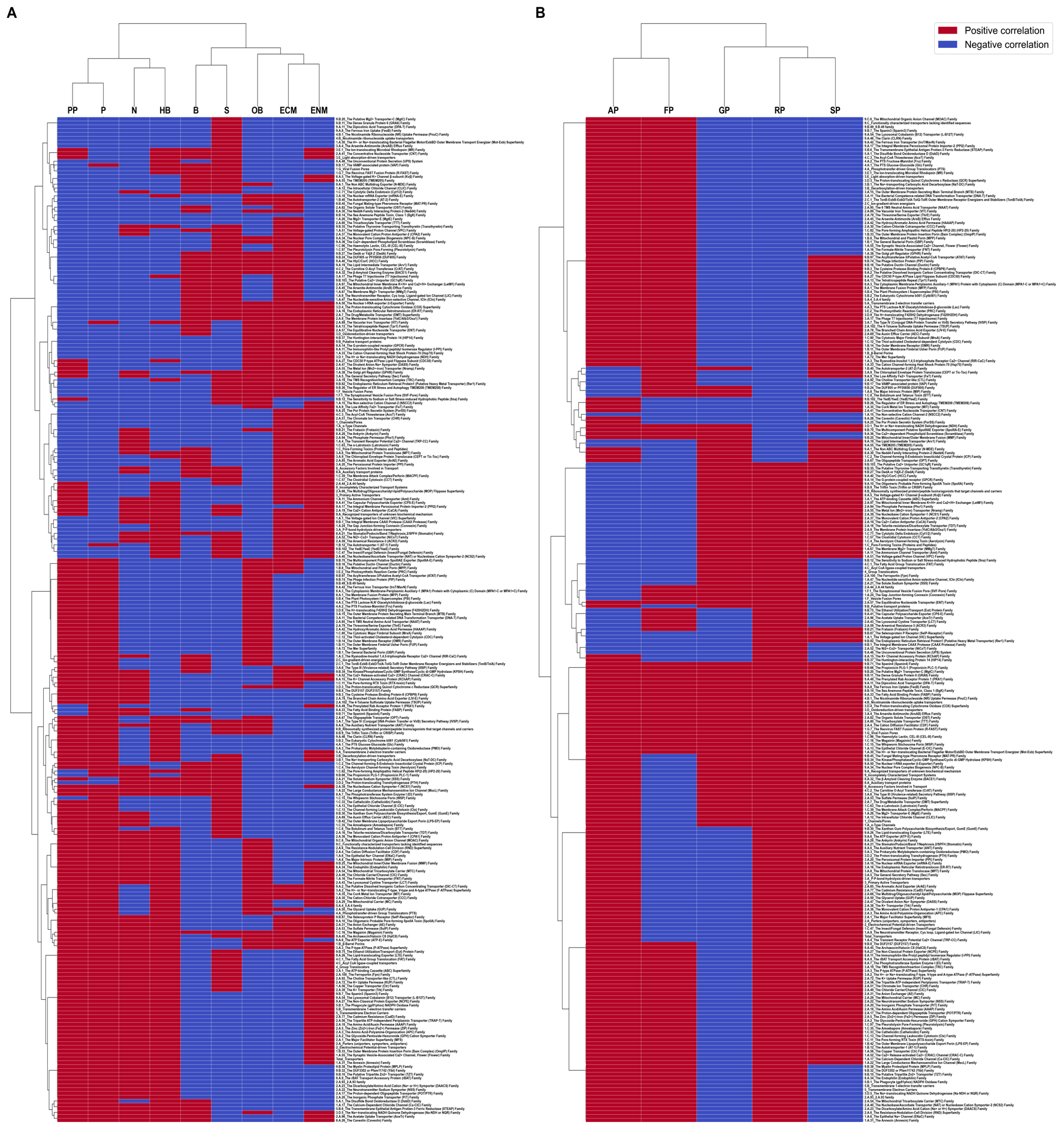
**

**Figure S5.** Clustered heatmap of the transcription factor (TF) families associated with DendroNet’s predictions of phytopathogenic lifestyles and traits. Also included is the ‘Total_TransFactors’ feature, which is the total number of TF genes annotated in each genome regardless of family. The values in the heatmap are the correlation directions for each TF family input into DendroNet as a machine learning feature, which indicate whether an increase (positive correlation, red) or decrease (negative correlation, blue) in the number of genes in a family was associated with a lifestyle or trait. Abbreviations: S, saprotrophs; ECM, ectomycorrhizal; ENM: endomycorrhizal; P, pathogens; PP, plant pathogens; N, necrotrophs; HB, hemibiotrophs; B, biotrophs; OB, obligate biotrophs; AP, angiosperm pathogens; GP, gymnosperm pathogens; FP, foliar pathogens; SP, stem pathogens; RP, root pathogens.

**
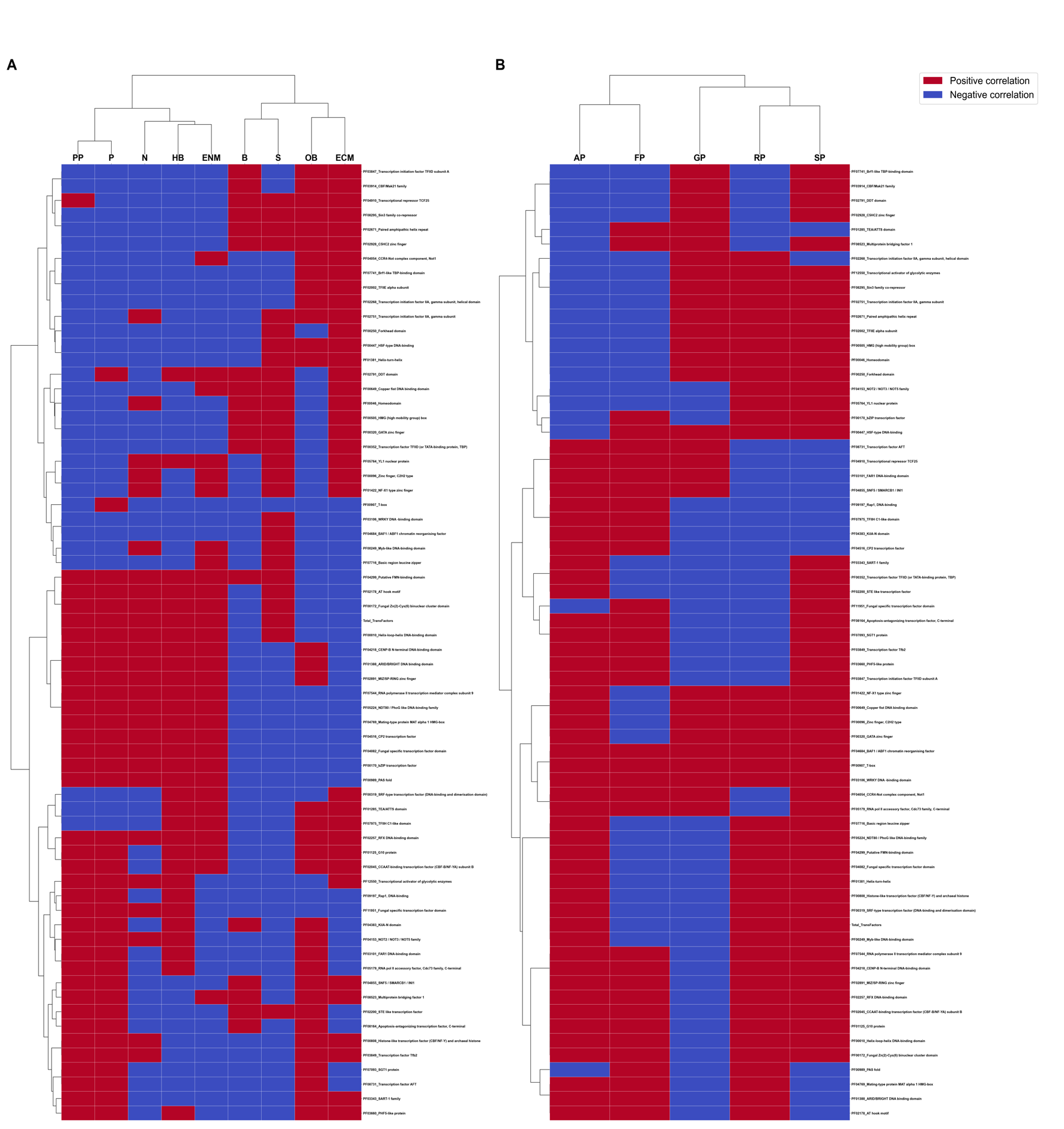
**

# Supplementary Data Descriptions

**Supplementary Data S1.** Reduced version of FunLifeDB with the 387 genomes (355 species) used for analyses in this study. Also included are the raw annotation data extracted from MycoCosm for carbohydrate active enzymes, peptidases, secondary metabolite clusters, transporters, and transcription factors. These data were used as input for both the principal component analyses and the DendroNet machine learning analyses.

**Supplementary Data S2.** Area under the receiver operating characteristic curve (AUC) scores obtained with DendroNet for the parsimony models (phylogenetic placement only, no genome annotation data) and feature+parsimony models (includes phylogenetic placement and genome annotation data) from all 31 feature sets for all lifestyles and phytopathogenic traits tested. The mean and standard deviation of the AUCs from each feature set were calculated, and the results for the feature+parsimony models are listed from highest mean to lowest mean AUC.

**Supplementary Data S3.** Results from all statistical analyses on DendroNet AUCs for both the lifestyles and phytopathogenic traits.

**Supplementary Data S4.** DendroNet feature importances from the feature+parsimony models for all five genome annotation groups. The raw, fraction, and correlation values for the individual genomic features (gene families) that improved DendroNet’s predictive performance of a lifestyle or trait over the parsimony model are reported for each annotation. A fraction value greater than 1.0 for a feature indicates that a DendroNet model using only that individual feature produced better AUC scores than a model using a combination of all features from an annotation group.

**Supplementary Data S5.** Correlation directions for all genomic features (gene families) included in the DendroNet feature+parsimony models, regardless of prediction performance relative to the parsimony model, for all five genome annotation groups. A positive correlation (+) indicates that a lifestyle or trait was associated with an increased number of genes relative to other lifestyles and traits, and a negative correlation (-) indicates that a lifestyle or trait was associated with a decreased number of genes.

**Supplementary Data S6.** A pruned, Newick format version of the phylogenetic tree downloaded from MycoCosm for input into DendroNet models. This reduced tree shows the phylogenetic relationships between the 387 fungal genomes analysed in this study.
